# Hyaluronan coating improves liver engraftment of transplanted human biliary tree stem/progenitor cells

**DOI:** 10.1186/s13287-017-0492-7

**Published:** 2017-03-20

**Authors:** Lorenzo Nevi, Guido Carpino, Daniele Costantini, Vincenzo Cardinale, Olga Riccioni, Sabina Di Matteo, Fabio Melandro, Pasquale Bartolomeo Berloco, Lola Reid, Eugenio Gaudio, Domenico Alvaro

**Affiliations:** 1 Department of Medico-Surgical Sciences and Biotechnologies, Sapienza University of Rome, Rome, Italy; 2 Department of Movement, Human and Health Sciences, Division of Health Sciences, University of Rome “Foro Italico”, Rome, Italy; 3 Department of Anatomical, Histological, Forensic Medicine and Orthopedics Sciences, Sapienza University of Rome, Rome, Italy; 4 Department of General Surgery and Organ Transplantation, Sapienza University of Rome, Rome, Italy; 5 Department of Cell Biology and Physiology and Program in Molecular Biology and Biotechnology, University of North Carolina School of Medicine, Chapel Hill, NC USA; 6 Department of Medicine and Medical Specialties, Sapienza University of Rome, Rome, Italy; 7 Division of Gastroenterology, Department of Medico-Surgical Sciences and Biotechnologies, Polo Pontino, Fondazione Eleonora Lorillard Spencer Cenci, Sapienza University of Rome, Vialedell’Università 37, 00185 Rome, Italy; 8 Division of Human Anatomy, Department of Anatomical, Histological, Forensic Medicine and Orthopedics Sciences, Sapienza University of Rome, Via Borelli 50, 00161 Rome, Italy

**Keywords:** Adult stem cells, Cell transplantation, Hepatocyte differentiation, Hyaluronan, Liver

## Abstract

**Background:**

Cell therapy of liver diseases with human biliary tree stem cells (hBTSCs) is biased by low engraftment efficiency. Coating the hBTSCs with hyaluronans (HAs), the primary constituents of all stem cell niches, could facilitate cell survival, proliferation, and, specifically, liver engraftment given that HAs are cleared selectively by the liver.

**Methods:**

We developed a fast and easy method to coat hBTSCs with HA and assessed the effects of HA-coating on cell properties in vitro and in vivo.

**Results:**

The HA coating markedly improved the viability, colony formation, and population doubling of hBTSCs in primary cultures, and resulted in a higher expression of integrins that mediate cell attachment to matrix components. When HA-coated hBTSCs were transplanted via the spleen into the liver of immunocompromised mice, the engraftment efficiency increased to 11% with respect to 3% of uncoated cells. Notably, HA-coated hBTSC transplantation in mice resulted in a 10-fold increase of human albumin gene expression in the liver and in a 2-fold increase of human albumin serum levels with respect to uncoated cells. Studies in distant organs showed minimal ectopic cell distribution without differences between HA-coated and uncoated hBTSCs and, specifically, cell seeding in the kidney was excluded.

**Conclusions:**

A ready and economical procedure of HA cell coating greatly enhanced the liver engraftment of transplanted hBTSCs and improved their differentiation toward mature hepatocytes. HA coating could improve outcomes of stem cell therapies of liver diseases and could be immediately translated into the clinic given that GMP-grade HAs are already available for clinical use.

**Electronic supplementary material:**

The online version of this article (doi:10.1186/s13287-017-0492-7) contains supplementary material, which is available to authorized users.

## Background

Liver transplantation is the only therapeutic option for many congenital and acquired liver diseases [1–3]. However, the requirements of this procedure and the paucity of organs available for transplantation limit this option to a minority of patients [1–3]. Cell therapy is a promising treatment strategy [1, 2, 4] and in this regard different cell sources have been tested, including hepatic stem cells (HpSCs), biliary tree stem cells (BTSCs), mesenchymal stem cells, adipose-derived stem cells, umbilical cord cells, amniotic fluid-derived epithelial cells, embryonic stem cells (ESCs), and induced pluripotent stem (iPS) cells [1, 2].

Recently, we successfully transplanted patients with advanced cirrhosis by infusing human biliary tree stem cells (hBTSCs) into the hepatic artery [1]. However, the homing and the liver engraftment of administered cells remain a key issue. Transplantation of such stem cell populations via the portal vein results in less than 5% engraftment [3, 5, 6]. Direct injection into the liver parenchyma results in ~10–20% engraftment [7]. The highest engraftment observed for transplantation of stem cells into the liver via a vascular route was reported by Khan et al. [8] who administered the cells via the hepatic artery. These findings were corroborated by others [9]. In addition, transplantation by any vascular route, either portal vein, hepatic artery, or spleen, resulted in significant ectopic cell distribution to the vascular beds of all tissues assayed. Moreover, the cells survived for months, increasing the risk of ectopic liver formation with unknown clinical consequences [7]. There is therefore a need for methods to increase localization of the transplanted cells to the target tissue.

Coinjection of human HpSCs with hyaluronans (HAs) triggered to form HA hydrogels into the livers of immunocompromised mice resulted in essentially 100% engraftment, an absence of ectopic cell distribution, and an extent of humanization of host livers at 2 weeks comparable with that at 3–5 months if transplanted without HA [7]. The use of natural and synthetic scaffolds has been considered by many [3, 7, 9–12].

HAs are of especial interest for stem cell therapies. They are polymers of the glucuronic acid–*N*-acetyl glucosamine dimer, are anionic, are nonsulfated, and are major constituents of all stem cell niches. HAs can be prepared as liquids that can be converted into hydrogels with remarkable chemical and mechanical properties ideal for cell populations, having perfect elasticity and enabling interstitial fluids complete access throughout the gel [11]. In addition, diverse forms of HA receptors, CD44 isoforms, are generic biomarkers of all known stem cell populations [13]. HAs, both in liquid and hydrogel forms, have been shown to promote stem cell survival and proliferation, to optimize spheroid/organoid formation of normal and transformed stem cells, and to optimize cryopreservability of stem cells [7, 11, 14–17]. They have also demonstrated promise in cell delivery [7, 18–21]. It is noteworthy that HA is renowned for fostering vascularization, and is readily modified by the cells as needed for integration into the tissue and with enzymes (e.g., hyaluronidases) and by hydrolysis native to the liver [7, 11, 18–21].

As a novel strategy to improve the liver engraftment of hBTSCs we are proposing to coat hBTSCs with HAs and to use them for cell therapy of liver diseases. We hypothesize that this strategy could be successful for the liver given that HA is selectively and specifically cleared by the liver [22, 23]. Moreover, HAs are largely used for different clinical applications, facilitating their use also in the setting of cell therapy [12]. The main objectives of the present study were to evaluate the effect of HA coating: in vitro, on engraftment, proliferation, and differentiation of hBTSCs in primary cultures; and in vivo, on engraftment efficiency into the liver and differentiation toward mature hepatocytes of hBTSCs transplanted, via the spleen, into immune-compromised mice.

## Methods

### Human tissue sourcing

The cell source consisted of freshly isolated human biliary tree stem/progenitor cells (hBTSCs) obtained from the human extrahepatic biliary tree, comprising the common hepatic duct, bile duct, cystic duct, gallbladder, and hepato-pancreatic ampulla. Biliary tissues were obtained from organ donors because they were not used for transplantation at the “Paride Stefanini” Department of General Surgery and Organ Transplantation, Sapienza University of Rome, Rome, Italy. Informed consent to use tissues for research purposes was obtained from our transplant program. All samples derived from adults between the ages of 19 and 73 years. Protocols received the approval of our Institutional Review Board, and processing was compliant with current Good Manufacturing Practice (cGMP). The research protocol was reviewed and approved by the Ethic Committee of Umberto I University Hospital, Rome, Italy. Freshly isolated hBTSCs have been characterized extensively in previous reports [24–26]. In the present report, the in-vitro experiments were performed by culturing primary freshly isolated cells, which were not passaged. Each set of experiments was performed on cells isolated from five different patients, in triplicate for each patient.

### Tissue processing

Tissue specimens were processed as described previously [7, 24, 25, 27, 28]. In brief, tissues were digested in RPMI 1640 supplemented with 0.1% human serum albumin, 1 nM selenium, antibiotics, type I collagenase (300 collagen digestion units/ml), 0.3 mg/ml deoxyribonuclease, at 37 °C with frequent agitation for 30–45 min.

### Epithelial cell adhesion molecule sorting procedures

Cells were sorted for epithelial cell adhesion molecule (EpCAM) using magnetic beads as indicated by the manufacturer (MiltenyiBiotec Inc., Germany). Briefly, the EpCAM+ cells were magnetically labeled with EpCAM MicroBeads (catalog #130-061-101; MiltenyiBiotec Inc.) and loaded onto a MACS LS Column (catalog #130-042-401; MiltenyiBiotec Inc.) that was placed in the magnetic field of a MACS Separator. EpCAM+ cells were suspended in basal medium at a concentration of 300,000 cells/ml, and used as the final cell suspension. Sorted cells were characterized extensively by FACS analysis for EpCAM and many mesenchymal cell markers (CD45, CD31, CD34, CD90, α-SMA).

### Media and solutions

All media were sterile filtered (0.22-μm filter) and kept in the dark at 4 °C before use. RPMI-1640, the basal medium used for all of the cell cultures, and fetal bovine serum (FBS) were obtained from GIBCO/Invitrogen (Carlsbad, CA, USA). All reagents were obtained from Sigma (St. Louis, MO, USA) unless otherwise specified.

### Kubota’s Medium

Kubota’s Medium (KM) is a serum-free medium developed for survival and expansion of endodermal stem/progenitors [29] and subsequently shown to be successful with human HpSCs [30, 31], hBTSCs [24, 26, 28, 32], human pancreatic stem/progenitor cells [28], and rodent HpSCs [14]. Mature endodermal cells do not survive in KM [33].The detailed protocol of its preparation was first reported by Kubota and Reid [29]. Briefly, KM consists of any basal medium (here being RPMI 1640) with no copper, low calcium (0.3 mM), 10^–9^ M selenium, 4.5 mM nicotinamide, 0.1 nM zinc sulfate heptahydrate, 10^–8^ M hydrocortisone (or dexamethasone), 5 μg/ml transferrin/Fe, 5 μg/ml insulin, 10 μg/ml high-density lipoprotein, 0.1% human (or bovine) serum albumin (HSA or BSA), and a mixture of purified free fatty acids that are added bound to purified HSA. The medium is now available commercially through PhoenixSongs Biologicals (Branford, CT, USA).

### Coating by hyaluronic acid

HA gel was made using HA powder (#S0780000; Sigma) dissolved in normal saline with a rate of 0.1% W/V and filtered by 0.22 μm. hBTSCs were counted by trypan blue, suspended with coating medium, mixed softly, and stored in an incubator for 10 min at 37 °C and 5.0 CO_2_. After 5 min in coating medium, hBTSCs were mixed softly.

### Cell cultures and clonal expansion

EpCAM+ hBTSCs (approximately 3 × 10^5^ cells), HA-coated or uncoated, were seeded onto 3-cm-diameter plastic culture dishes and kept overnight (~12 hours) in KM with 10% FBS. Thereafter cell cultures were maintained in serum-free KM. For testing the clonal expansion of hBTSCs, a single cell suspension was obtained, and the cells were plated on culture plastic at a clonal seeding density (500/cm^2^) [34]. For in-vitro experiments, cells were cultured in serum-free KM (without passing).

### Cell viability

Cell viability was determined by trypan blue exclusion assay (#302 643-25G; Sigma). The cells stained blue were dead; the viable cells did not stain. This dye was used at 1:1 v/v with the cell buffer. The cell count was carried out using FAST-READ 102 (#BSV100; Biosigma). Cells viability was calculated at four different time points: 1, 3, 7, and 14 days.

### Colony counting

The hBTSC colonies began to appear after 1 day of plating and were easily identified by observing at 10× with a light microscope. Any size colony was counted as one, whether large ones at >3000 cells or small ones at <200 cells. Each well of the six well plates was evaluated using 10× magnification for colonies and counted during 2 weeks of culture. Microscopic fields were used as normalization. Observations of colony number, size, and morphology were noted.

### Population doubling

The proliferation rate was analyzed on the same hBTSC population, seeded in six multiwell plates at a density of 1 × 10^4^ cell/cm^2^, and cultured for 7 days. The cell counts were performed under two culture conditions: hBTSCs coated with HA; and hBTSCs uncoated.

The medium was changed every 3 days, using serum-free KM. The mean cell number was calculated on three experimental samples for each condition, and cell density was expressed as the mean of cells/cm^2^ ± standard deviation (SD). Cells were detached from supports and were counted by trypan blue assay; for these experiments we used only viable cells.

The population doubling time (PDT) was calculated in the phase exponential growth by the following formula [35]:

$$ P D T= l o{g}_{10} x\varDelta T/ l o{g}_{10}\left({N}_{7d}\right)\mathit{\hbox{-}} l o{g}_{10}\left({N}_{1d}\right) $$

where *N*_7d_ is the cell number at day 7 and *N*_1d_ is the cell number at day 1.

To determine the population doubling (PD) rate, the following formula was applied [35]:

$$ P D= l o{g}_{10}(N)- l o{g}_{10}\left({N}_s\right)/ l o{g}_{10}(2) $$

where *N* is the harvested cell number and *N*_s_ is the initial plated cell number.

### Quantitative reverse-transcription polymerase chain reaction analysis

Total RNA was extracted by the procedures of Chomczynski and Sacchi [36]. The expression of the genes was conducted by reverse-transcription and PCR amplification performed in a closed tube (OneStep RT-PCR; Qiagen, Hamburg, Germany) on total RNA samples extracted from cells and tissues. These genes were coamplified with the GAPDH housekeeping gene as a reference. The mRNA expression was measured by the quantification of amplicons with on-chip capillary microelectrophoresis performed with the Experion System (Bio-Rad, UK). The expression of the gene of interest was calculated by the ratio of the concentrations of the gene of interest and the reference gene GAPDH “in vitro” and β-actin “in vivo” (reported by the instrument in nmol/L) (Additional file 1: Table S1).

### Immunofluorescence on cell cultures

For immunofluorescence (IF) on cell cultures, slide chambers were fixed in acetone for 10 min at room temperature and then rinsed with PBS-Tween 20. Nonspecific protein binding was blocked by 5% normal goat serum. Fixed cells were incubated with primary antibodies against HA (# BVN-3996 F-100; Vinci Biochem) and CD44 (# 3570; Cell Signaling Technology). Cells were then washed and incubated for 1 hour with labeled isotype-specific secondary antibodies (anti-mouse AlexaFluor-488, anti-rabbit AlexaFluor-594; Invitrogen, LifeTechnologies Ltd, Paisley, UK) and counterstained with 4,6′-diamidino-2-phenylindole (DAPI) for visualization of cell nuclei. For all immunoreactions, negative controls were also included and consisted of replacing the primary antibody with preimmune serum. Cultures were examined in a coded fashion by Leica Microsystems DM 4500 B Light and Fluorescence Microscopy (Weltzlar, Germany) equipped with a JenoptikProg Res C10 Plus Videocam (Jena, Germany). IF was also analyzed by confocal microscopy (Leica TCS-SP2) [25].

### Cell transplantation in severely combined immunodeficient mice

All animal experiments were carried out in accordance with the EU Directive 2010/63/EU for animal experiments and with Sapienza institutional guidelines. The severely combined immunodeficient (SCID) mice (T/SOPF NOD.CB17 PRKDC/J) were male, 4-week-old animals and were used as the hosts for transplantation of human cells. Animals were sedated with an anesthetic drug (2, 2, 2-tribromoethanol). Thereafter, 2 × 10^6^ hBTSCs coated with HA or uncoated (control cells) were suspended in 100 μl saline and injected into the liver via the spleen. Sham controls were infused only with 100 μl saline or with HA buffer 0.1% w/v. Then, 30 days after cell transplantation, mice were sacrificed and the livers removed for further analyses. Liver samples were placed in Trizol reagent for gene analyses or in 4% formalin for pathologic and immunohistochemistry (IHC) analyses. Blood samples were collected from the heart, centrifuged, and serum samples stored at –20 °C for quantification of human albumin by enzyme-linked immunosorbent assay (ELISA) (Albumin Human ELISA Kit, cat. N. ab108788; Abcam, Cambridge, UK).

### Light microscopy and immunohistochemistry

Specimens were fixed in 10% buffered formalin for 2–4 hours, embedded in low-temperature-fusion paraffin (55–57 °C), and 3–4 μm sections were stained with hematoxylin–eosin and Sirius red/Fast green, according to standard protocols. For IHC, endogenous peroxidase activity was blocked by 30-min incubation in methanolic hydrogen peroxide (2.5%). Antigens were retrieved, as indicated by the vendor, by applying Proteinase K (code S3020; Dako, Glostrup, Denmark) for 10 min at room temperature. Sections were then incubated overnight at 4 °C with primary antibodies (Additional file 2: Table S2). Samples were rinsed twice with PBS for 5 min, incubated for 20 min at room temperature with secondary biotinylated antibody (LSAB+ System-HRP, code K0690; Dako) and then with Streptavidin-HRP (LSAB+ System-HRP, code K0690; Dako). Diaminobenzidine (Dako) was used as a substrate, and sections were counterstained with hematoxylin or with Periodic Acid–Schiff (PAS). For all immunoreactions, negative controls were included by replacing the primary antibody with preimmune serum. Sections were examined in a coded fashion by Leica Microsystems DM 4500 B Light and Fluorescence Microscopy (Weltzlar, Germany) equipped with a JenoptikProg Res C10 Plus Videocam (Jena, Germany). Observations were processed with an Image Analysis System (IAS; Delta Sistemi, Rome, Italy) and were independently performed by two pathologists in a blind fashion. A list of positive and negative controls is presented in Additional file 3: Table S3.

For IF, nonspecific protein binding was blocked with 5% normal goat serum. Sections were incubated with primary antibodies, and subsequently incubated with labeled isotype-specific secondary antibodies (anti-mouse AlexaFluor-488 and anti-rabbit Alexafluor-594; Invitrogen Ltd) for 1 hour; nuclei were visualized with DAPI [25]. To perform double immunostaining with two mouse primary antibodies, we followed a three-step protocol: sections were incubated with the first primary antibody, an anti-mouse (or anti-rabbit) secondary fluorescent antibody (alexafluor-488) was applied, and the second primary antibody was prelabeled with a fluorophore using the APEX-594 labeling kit (Invitrogen) and applied to the section. All antibodies were diluted (1:50) and incubated for 1 hour. Slides were counterstained with DAPI. For all immunoreactions, adequate negative controls were also preformed [25].

All counts were performed in six nonoverlapping fields (magnification 20×) for each slide; at least three different slides were taken from each specimen. For IHC/IF staining, the number of positive cells was counted in a random, blinded fashion in six nonoverlapping fields (magnification 20×) for each slide/culture, and the data are expressed as % positive cells. The hBTSC engraftment in murine livers and their differentiation was assessed by IHC for anti-human antibodies (anti-human mitochondria) which do not react with mouse antigens, as described elsewhere [25].

### Flow cytometry analysis

Isolated cells were labeled with fluorescent primary antibodies or adequate isotype controls. Cells were resuspended at approximately 2 × 10^5^ cells/ml in PBS. Primary antibodies included EpCAM (EpCAM-FITC, catalog #130-080-301; MiltenyiBiotec Inc.), and many mesenchymal cell markers (CD45, CD31, CD34, CD90, α-SMA) (detailed information presented in Additional file 2: Table S2). Cells were analyzed by a BD FACScanto™ Flow Cytometer (Becton, Dickinson and Company, NJ, USA). Ten thousand events were acquired and analyzed by BD FACSDiva™ software (Becton, Dickinson and Company).

### Statistical analysis

Data were expressed as mean ± SD. Statistical analyses were performed by SPSS statistical software (SPSS Inc., Chicago IL, USA). Differences between groups for nonnormal distribution parameters were tested by Mann–Whitney *U* tests. Statistical significance was set to *p* < 0.05.

## Results

### HA coating

Immediately after the isolation, hBTSCs were incubated with 0.1% HA (# S0780000; Sigma) in KM (0.1% weight/volume) and gently mixed for 10 min. The cells were then cultured in serum-free KM.

**Fig. 1:**
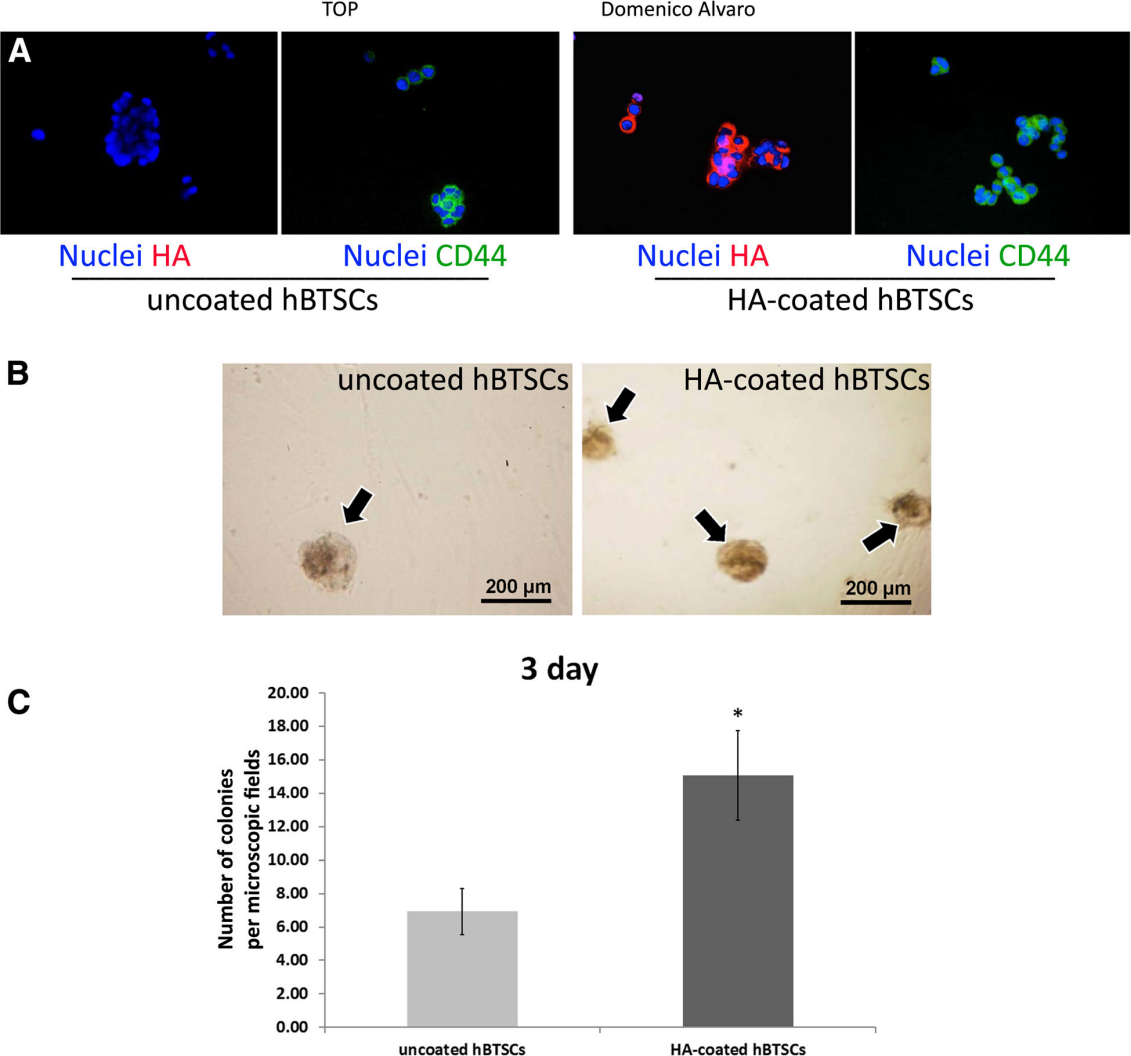
Successful hyaluronan (*HA*) coating. **a** HA coating. IF analysis of HA in uncoated (*left panels*) and HA-coated (*right panels*) human biliary tree stem cells (*hBTSCs*) demonstrates expression of HA only in hBTSCs, which underwent a coating procedure (*red* dye; nuclei, *blue* dye) (40× magnification). IF analysis of CD44 in uncoated (*left panels*) and HA-coated (*right panels*) hBTSCs demonstrates expression of CD44 in hBTSCs (CD44, *green* dye; nuclei, *blue* dye) (40× magnification). This image is representative of the findings from *n* = 5 experiments. **b** Colony counting at day 3 in cultures of HA-coated and uncoated hBTSCs. Optical microscope contrast phase appearance of cultures of HA-coated and uncoated hBTSCs. This image is representative of the findings from *n* = 5 experiments (magnification 10×). **c** Histograms represent the number of colonies. Data expressed as mean ± SD of *n* = 3 different experiments; *p < 0.00001

After the coating procedure, cells were washed twice with KM and observed by optical microscope. The HA coating was evaluated by IF using an anti-HA antibody (code PAA182Ge01; Cloud-Cclone Corp.) (Fig. 1a). The expression of the HA receptor, CD44, was verified in hBTSCs, coated and uncoated, as assayed by IF using human CD44 specific antibody (mouse anti-CD44, N#3570; Cell Signalling, Danvers, MA, USA) (Fig. 1a). As shown in Fig. 1a, HA—marked by the specific antibody—uniformly covered hBTSCs, which resulted in cells constantly positive for CD44. The procedure of cell coating resulted in the formation of cell clusters (2–5 cells) with a diameter ranging from 12 to 19 μm. The formation of cell clusters (>2 cells) was significantly higher in HA-coated versus uncoated hBTSCs (*p* < 0.05; *N* = 5).

Colony formation is a well-established parameter of cell viability and attachment. Isolated HA-coated and uncoated hBTSCs were plated onto culture plastics and in KM at a density of 10,000 cells/ml, and after 3 days in culture the colonies were counted (Fig. 1b, c). A significantly higher number of cell colonies was present in HA-coated as compared with uncoated hBTSCs (uncoated hBTSCs: 6.93 ± 1.39; HA-coated hBTSCs: 15.07 ± 2.69; *p* < 0.001; *N* = 3) (Fig. 1b, c).

### Effects of HA coating on hBTSC viability, proliferation, and gene expression in vitro

**Fig. 2:**
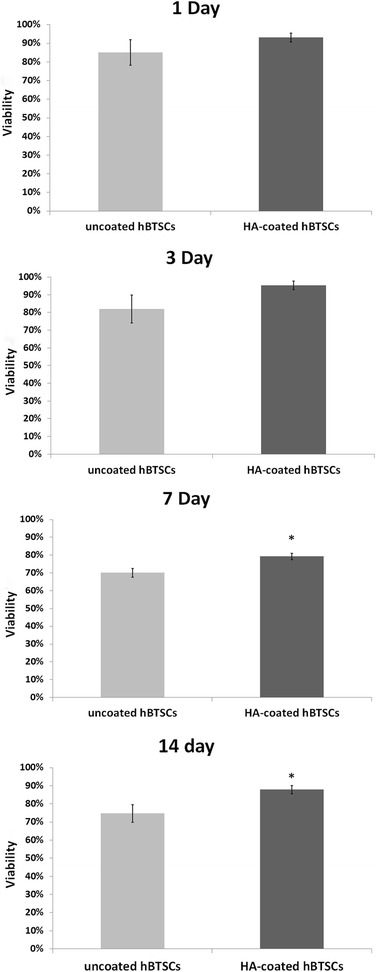
Cell viability of hyaluronan (*HA*)-coated and uncoated human biliary tree stem cells (*hBTSCs*) at different times in cultures. After 7 and 14 days in culture, viability (trypan blue exclusion) was better in HA-coated as compared with uncoated hBTSCs. Data expressed as mean ± SD for three experiments; *p < 0.05

Viability of HA-coated and uncoated control cells was assessed (trypan blue exclusion) at different time points (i.e., after their initial isolation or after 1, 3, 7, or 14 days in culture). After their isolation and at days 1 and 3, viability was similar between HA-coated and uncoated hBTSC cultures (isolation: uncoated 90.12 ± 6.83%, HA-coated 94.03 ± 2.32; day 1: uncoated 85.12 ± 6.83%, HA-coated 93.03 ± 2.32; day 3: uncoated 95.27 ± 2.47%, HA-coated 82.01 ± 7.92%; *N* = 3) (Fig. 2). By days 7 and 14, HA-coated hBTSCs cells showed an increased viability with respect to control uncoated cells (day 7: uncoated hBTSCs 70.06 ± 2.40%, HA-coated hBTSCs 79.24 ± 1.83%; *p* < 0.05; day 14: uncoated hBTSCs 74.73 ± 4.88%, HA-coated hBTSCs 87.83 ± 2.30; *p* < 0.05; *N* = 3, Fig. 2).

**Fig. 3:**
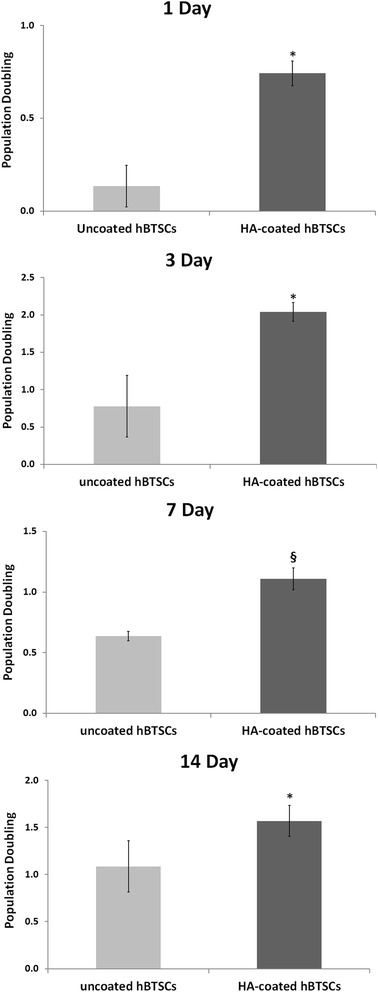
Population Doubling (PD) of uncoated versus hyaluronan (*HA*)-coated human biliary tree stem cells (*hBTSCs*) at different times in culture. PD was always higher in HA-coated cells as compared with uncoated hBTSCs. Data expressed as mean ± SD for three experiments; *p < 0.05, §*p* < 0.01

The proliferation rate, evaluated by the PD method, was markedly higher for HA-coated hBTSC cultures than for the uncoated controls at all of the different times of observation (day 1: 0.74 ± 0.06 vs 0.13 ± 0.11; *p* < 0.05; *N* = 3; day 3: 2.03 ± 0.12 vs 0.77 ± 0.41; *p* < 0.05; *N* = 3; day 7: 1.10 ± 0.09 vs 0.63 ± 0.03; *p* < 0.01; *N* = 3; day 14: 1.95 ± 0.03 vs 0.94 ± 0.22; *p* < 0.05; *N* = 3; Fig. 3). The gene expression of different adhesion molecules was analyzed by quantitative reverse-transcription polymerase chain reaction (RT-qPCR) in primary cultures of hBTSCs under KM conditions, which were extensively characterized by FACS analysis for EpCAM and many mesenchymal cell markers (CD45, CD31, CD34, CD90, α-SMA) (data not shown).

**Fig. 4:**
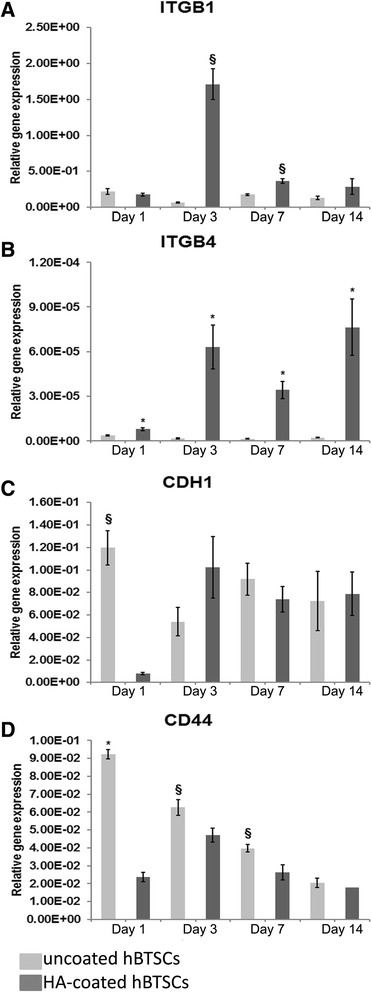
Expression of adhesion molecule genes in culture of hyaluronan (*HA*)-coated and uncoated human biliary tree stem cells (*hBTSCs*). **a** Gene expression of integrin subunit beta 1 (*ITGβ1*) in HA-coated hBTSCs (*dark gray columns*) was higher (days 3 and 7) than uncoated hBTSCs (*light gray columns*). Data expressed as mean ± SD of *n* = 5 experiments; §*p* < 0.01. **b** Gene expression of integrin subunit beta 4 (*ITGβ4*) in HA-coated hBTSCs was higher (days 1, 3, 7, and 14) than in uncoated hBTSCs. Data expressed as mean ± SD of *n* = 5 experiments; *p < 0.05. **c** Gene expression of E-cadherin (*CDH1*) in uncoated hBTSCs was higher (day 1) than in HA-coated hBTSCs. Data expressed as mean ± SD of *n* = 5 experiments; §*p* < 0.01. **d** Gene expression of CD44 in uncoated hBTSCs was higher (days 1, 3, and 7) than in HA-coated hBTSCs. Data expressed as mean ± SD of *n* = 5 experiments; *p < 0.05, §*p* < 0.01

HA-coated cells, as compared with uncoated cells, had enhanced gene expression of integrin subunit beta 1 (ITGβ1) (day 3: 1.71 × 10^0^ ± 2.14 × 10^–1^ vs 6.65 × 10^–2^ ± 1.02 × 10^–2^; *N* = 5; *p* < 0.01; day 7: 3.69 × 10^–1^ ± 3.24 × 10^–2^ vs 1.78 × 10^–1^ ± 1.26 × 10^–2^; *N* = 5; *p* < 0.01; Fig. 4a) and integrin subunit beta 4 (ITGβ4) (day 1: 7.93 × 10^–6^ ± 9.16 × 10^–7^ vs 3.57 × 10^–6^ ± 3.57 × 10^–7^; *N* = 5; *p* < 0.05; day 3: 6.30 × 10^–5^ ± 1.48 × 10^–5^ vs 1.50 × 10^–6^ ± 4.32 × 10^–7^; *N* = 5; *p* < 0.05; day 7: 3.43 × 10^–5^ ± 5.75 × 10^–6^ vs 1.43 × 10^–6^ ± 1.14 × 10^–7^; *N* = 5; *p* < 0.05; day 14: 7.63 × 10^–5^ ± 1.89 × 10^–5^ vs 2.33 × 10^–6^ ± 2.74 × 10^–7^; *N* = 5; *p* < 0.05; Fig. 4b). By contrast, HA-coated cells, when compared with uncoated cells, showed a decrease gene expression of cadherin-1 (CDH1) (day 1: 7.99 × 10^–3^ ± 8.33 × 10^–4^ vs 1.20 × 10^–1^ ± 1.53 × 10^–2^; *N* = 5; *p* < 0.01; Fig. 4c) and CD44 (day 1: 2.36 × 10^–2^ ± 2.66 × 10^–3^ vs 9.24 × 10^–2^ ± 1.31 × 10^–2^; *N* = 5; *p* < 0.05; day 3: 4.71 × 10^–2^ ± 4.39 × 10^–3^ vs 6.25 × 10^–2^ ± 2.60 × 10^–3^; *N* = 5; *p* < 0.01; day 7: 2.62 × 10^–2^ ± 2.17 × 10^–3^ vs 3.97 × 10^–2^ ± 3.95 × 10^–3^; *N* = 5; *p* < 0.01; Fig. 4d).

### Engraftment of HA-coated (vs uncoated) hBTSCs after intrasplenic transplantation in mice

**Fig. 5:**
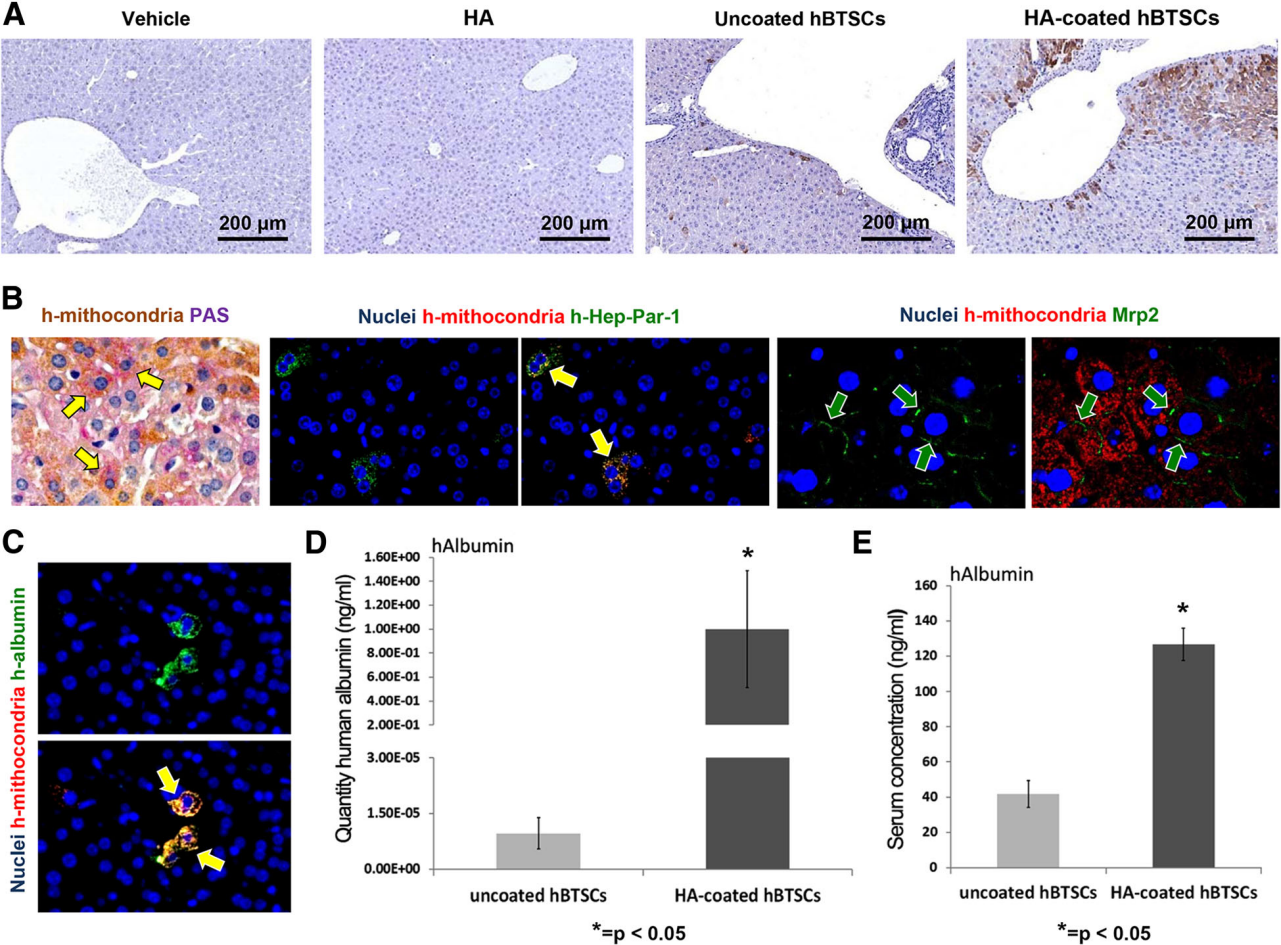
In-vivo engraftment of human biliary tree stem cells (*hBTSCs*) after intrasplenic transplantation in mice. **a** Four weeks after hBTSC injection into the spleen, the livers were analyzed by immunohistochemistry (IHC) utilizing anti-human (*h*) mitochondria. Expression of human mitochondria antigen in liver parenchyma of SCID mice indicated that a higher proportion of liver murine parenchyma was repopulated by human cells in mice transplanted with HA-coated hBTSCs in comparison with mice injected with uncoated cells. No positive cells have been found in control groups. **b** Signs of differentiation toward mature hepatocyte were revealed in anti-mitochondria positive cells within the livers of mice injected with HA-coated hBTSCs. In particular, human cells showed glycogen storage (PAS positivity, *arrows*), Hep-Par-1 expression (*arrow*), and the expression of multidrug resistance-associated protein 2 (*Mrp-2*) in a canalicular pattern. Original magnification (OM) = 40×. **c** Anti-human mitochondria positive cells within the livers of mice injected with HA-coated hBTSCs showed positivity for anti-human albumin (*arrows*). OM = 40×. **d** RT-qPCR expression of human albumin in the livers of mice transplanted with HA-coated versus uncoated hBTSCs. After 30 days, human albumin gene expression in the livers of mice transplanted with HA-coated cells was markedly higher than in mice transplanted with uncoated hBTSCs. **e** Human serum albumin in mice transplanted with HA-coated and uncoated hBTSCs. Human albumin concentration measured in the serum of mice transplanted with HA-coated hBTSCs was double that of mice transplanted with uncoated hBTSCs. *HA* hyaluronan, *PAS* Periodic Acid–Schiff

To determine whether HA-coated hBTSCs engraft and proliferate in mouse liver, cells were injected into the liver via the spleen of SCID mice and, after 4 weeks, the livers were analyzed by IHC utilizing an ultraspecific anti-human mitochondria antibody. As shown in Fig. 5a, uncoated and HA-coated hBTSCs engrafted into the murine liver parenchyma (*N* = 3). Specifically, the expression of human mitochondrial protein in liver parenchyma of the SCID mice indicated that 2.626 ± 1.53% and 11.015 ± 8.167% of parenchymal cell mass derived from uncoated or HA-coated hBTSCs respectively (*p* < 0.05). The quantitative analysis showed an increase (Fig. 5a) in humanized host parenchymal cell mass when the mice were transplanted with HA-coated hBTSCs as compared with uncoated cells (*N* = 3; *p* < 0.05), thus demonstrating enhanced engraftment of HA-coated hBTSCs. As expected, human cells were not present within the liver of SCID mice infused either with normal saline (vehicle, Fig. 5a) or with HA buffer with normal saline (vehicle, Fig. 5a) or with HA buffer (Fig. 5a). Interestingly, the study of mature hepatocyte markers revealed that human mitochondria positive cells showed signs of differentiation toward mature hepatocytes such as PAS positivity (glycogen storage), Hep-Par1 positivity, Albumin positivity, and Mrp1 positivity with a canalicular pattern expression.

At same time point, human albumin gene expression in the liver was evaluated as a quantitative measure for stem cell engraftment and as a sign of differentiation toward mature human hepatocytes. Interestingly, before transplantation into the host, both HA-coated and uncoated freshly isolated hBTSCs showed minimal or null mRNA albumin levels with respect to hBTSCs cultured in high defined medium for hepatocyte differentiation (HDM-H) or with respect to primary mature hepatocytes (Additional file 4: Figure S1). Human albumin expression was analyzed by RT-qPCR utilizing human and mouse specific primer sequences. Data have been normalized with β-actin as a housekeeping gene. In the liver samples from mice transplanted with HA-coated hBTSCs, human albumin gene expression was markedly higher than in mice treated with uncoated hBTSCs (1.00 ± 0.84 vs 0.000008 ± 7.29; *N* = 3; *p* < 0.05; Fig. 5b). As a consequence, the human albumin/mouse albumin ratio (ALB homo/ALB mouse) resulted in markedly higher levels in mice treated with HA-coated than uncoated hBTSCs (0.39 ± 0.31 vs 0.000036 ± 0.000014; *N* = 3; *p* < 0.01). Circulating serum human albumin was measured by ELISA in transplanted mice after 4 weeks from transplantation. Mice treated with HA-coated hBTSCs showed a concentration of serum human albumin that was approximately double that found in mice transplanted with uncoated hBTSCs (117.62 ± 18.20 vs 64.94 ± 15 ng/dl; *N* = 3; *p* < 0.05; Fig. 5c). Human albumin was undetectable in sham operated control mice by the ELISA assay we used (*n* = 3; Fig. 5c).

### Evaluation of ectopic distribution and seeding of transplanted cells

**Fig. 6:**
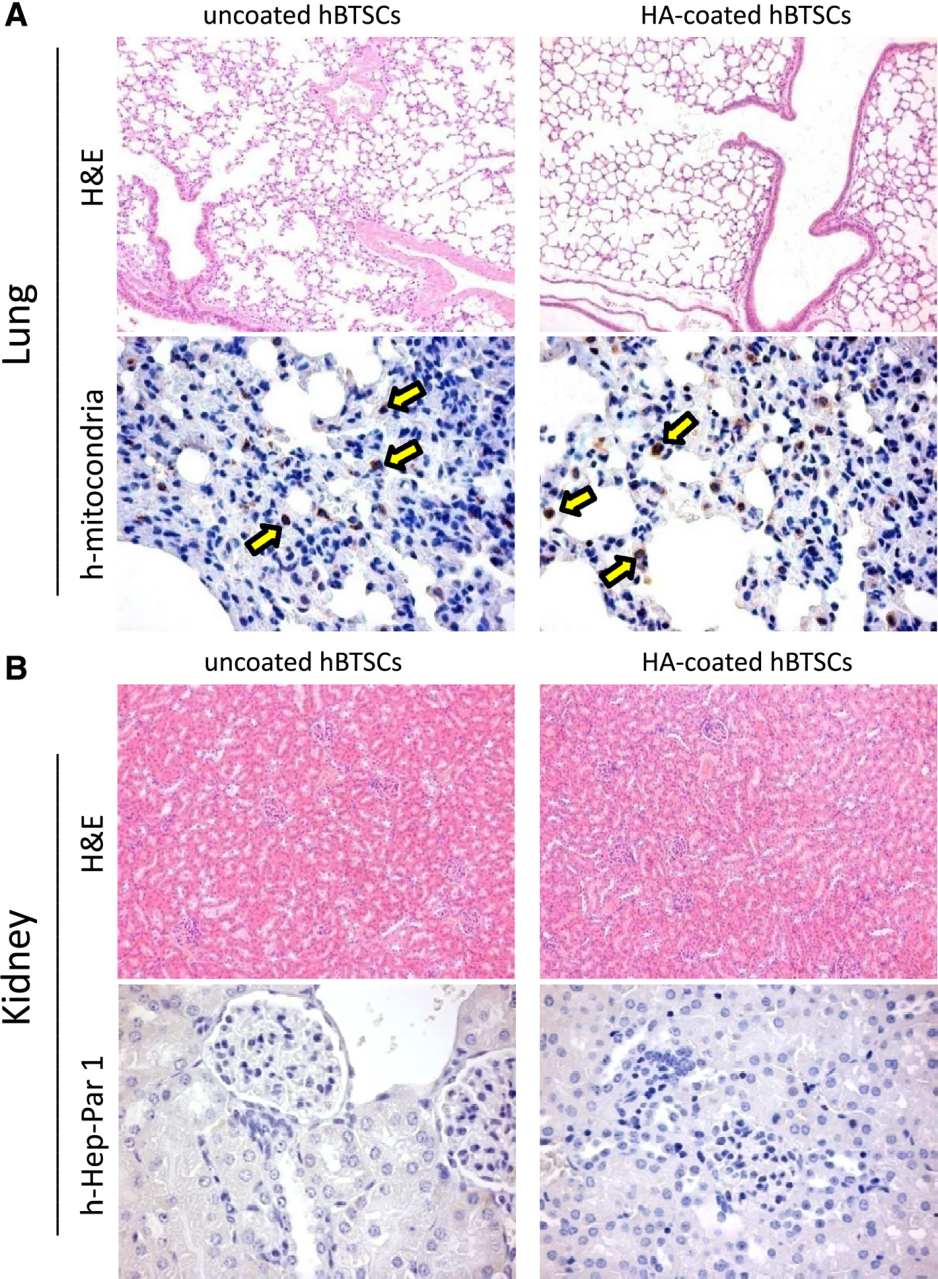
Histomorphological analysis of distant organs to assess ectopic cell distribution. The lung and the kidney were evaluated by histology and immunohistochemistry (IHC) to test the occurrence of ectopic distribution of human cell emboli. With respect to the lung (**a**), hematoxylin and eosin (*H&E*) staining revealed normal histomorphological aspects. Few and scattered human mitochondria positive cells (*arrows*) were observed, without differences in their numbers between animals transplanted with uncoated versus HA-coated hBTSCs (**a**). Also the kidney appeared invariably normal (**b**) with respect to the histomorphological aspects; no human Hep-Par1+ cells were identified by IHC. *HA* hyaluronan, *hBTSC* human biliary tree stem cells

To evaluate the possibility of ectopic distribution and seeding of transplanted cells, distant organs, the lung, and the kidney were analyzed for the occurrence of human cell emboli using histology and IHC. The lungs appeared invariably normal with respect to histomorphological aspects (*N* = 3; Fig. 6). By IHC, few and scattered human mitochondria positive cells (arrows) were identified; the extent of ectopic cell distribution was similar in mice transplanted with uncoated versus coated BTSCs (*N* = 3; Fig. 6). Also the kidney appeared invariably normal with respect to the histomorphological aspects (*N* = 3; Fig. 6) and, by IHC, no human Hep-Par1+ cells were seen (Fig. 6). However, the IHC using an antibody to human mitochondria resulted in staining of renal tubules (not shown) in transplanted mice (hBTSCs vs HA-coated hBTSCs) as well as in sham transplanted (control) mice. We speculate that this might be nonspecific staining (i.e., cross-contamination in staining). Finally, no tumoral mass has been observed in the abdomen, liver, spleen, kidney, lungs, or derma at sacrifice.

## Discussion

Coating of hBTSCs with hyaluronans (HA) resulted in significant improvement in engraftment of cells delivered by a vascular route to the liver. Many different strategies are currently under investigation to improve cell grafting, including the use of organic and inorganic biomatrices mimicking the microenvironment offered by the extracellular matrix [7, 37, 38]. HAs, major constituents of stem cell niches, are candidate coatings of stem cells used for cell therapies because they facilitate viability, proliferation, and engraftment in damaged livers. The chemical and mechanical properties of HA are conducive to essential requirements for stem cells. In addition, because the liver is a primary site for HA clearance, HA coating represents an advantageous strategy for the selective targeting of the transplanted cells to the liver. In our work, we tested the coating of hBTSCs with HA for its influence on engraftment into livers.

This molecule is already approved for Good Manufactory Practice (GMP) use and has been tested in clinical trials evaluating its effect on dental implant placement [39, 40], on treatment of arthritis after injections into joints [39], and for various types of cosmetic surgery [40].

A simple and rapid coating protocol was achieved and consisted of incubating cells in a 0.1% HA (weight/volume) solution for 10 min at room temperature. The IF analysis showed that HA was uniformly distributed around the whole cell surface, representing a homogeneous coating that was stable throughout the 2-week cultures. Previously, Roberts et al. [41] obtained similar findings but with polyethylene glycol (PEG).

A crucial issue in cell therapies is the size of transplanted cells. If the cells are large or if they form large aggregates, the transplantation of them via a vascular route can result in an embolus that can be life threatening. If the cells are small, their engraftment efficiency can be very low, and the cells will have a greater propensity to distribute to ectopic sites. Both possibilities are of importance for stem cell therapy considerations. Cells used for cell therapy of liver diseases have been infused into the liver via the spleen in animal models or into the portal vein or hepatic artery in humans. The sizes in terms of the cell diameters have ranged from ~8–10 μm for stem cells (ESCs, HpSCs, BTSCs), to ~12–15 μm for hepatoblasts and committed progenitors, to ~17–18 μm for diploid hepatocytes, found in abundance in neonatal livers, to ~25–30 μm for mature hepatocytes that are dominant in adult livers [2, 3, 6]. Engraftment efficiencies of less than 5% were reported for stem cells when delivered by vascular routes into the livers of primates [6] or in the livers of humans when injected into the portal vein [8]. Engraftment efficiencies were increased to 20–25% if delivered via the hepatic artery [8].

In these studies with HA coatings, the danger of thrombi generation has been minimized, because the coating process did not result in large cell clusters. The dimensions of HA-coated hBTSC aggregates (maximum of 3 cells) were within the range of previous studies with uncoated cells (<30 μm), and therefore their administration is hypothesized to be safe.

HA coatings resulted in significant improvements in hBTSC biological properties essential for transplantation and engraftment. Cell viability, colony formation capacity, and PD of HA-coated hBTSCs were better than uncoated cells, as verified in long-term cultures corroborating prior reports of the effects of HA on both normal cells [7, 11, 17, 28], in protecting cells under cryopreservation conditions [16], and in support of transformed cancer stem cells in vitro and in their survival and proliferation in vivo [15].

A facet of the ability of the HA coating to facilitate these biologic properties is its ability to maintain critical cell adhesion molecules needed for cell attachment and cell–cell interactions and to prevent them from internalization following cell suspension preparations or with transplantation [7]. We analyzed ITGβ1 (CD29) and ITGβ4 (CD104) integrins that bind, respectively, collagen type III and laminin, CD44, a primary receptor for HA, and, finally, CDH1 (also known as E-cadherin) which allows the formation of cell–cell bonds. A significant enhanced expression of the two integrins, ITGβ1 and ITGβ4, was observed in HA-coated cells and is consistent with the improved in-vitro properties and enhanced engraftment in vivo.

In light of the promising in-vitro findings, we next moved on to in-vivo experiments aimed at evaluating whether HA coating could improve the liver engraftment of hBTSCs. The intrasplenic infusion route was chosen, because it is largely utilized in preclinical studies with different cell types such as hBTSCs. The intrasplenic infusion delivers the cells via the portal vein, a route that routinely yields ~3% engraftment. Our data indicated that HA coating enhanced by 4-fold the engraftment efficiency without detectable spreading of the cells to the kidney and with no increased spread to the lungs over that observed with uncoated cells. In addition, the appearance of signs of differentiation toward mature cells was revealed in engrafted cells. Indeed, using RT-qPCR we demonstrated that, after 30 days, gene expression of human albumin in the mouse liver was markedly enhanced and, consistently, human serum albumin was doubled in mice transplanted with HA-coated vs uncoated hBTSCs. Our method of human albumin detection is highly sensitive, because our primers accurately discriminate between human and mouse albumin, minimizing the possibility of interference. Indeed, we designed primers by considering a specific adenine in the albumin gene sequence that allows a perfect discrimination between the human and murine albumin genes; this adenine avoids the annealing of the primer with the cDNA of murine albumin. Notably, the gene expression and the protein concentration of the albumin in the serum of mice are not directly linked. Different biologic processes involved in the production secretion, metabolism, and excretion of human albumin in the serum of the treated mice could account for this apparent discrepancy. The results concerning albumin secretion are in touch with the IHC observations which showed appearance of signs of differentiation toward mature cells rather than complete differentiation.

In substance, our results indicate that HA coating plays a key role in cell adhesion also in vivo. HA should create a favorable microenvironment allowing a greater number of hBTSCs to engraft within the liver parenchyma, survive, proliferate, and enhance human albumin secretion. When, in previous studies, fibrin was tested, instead of the HA, the same phenomenon was observed [6]. In favor of our proposal is the fact that HA is disposable in GMP grade for clinical use and, primarily, that it is selectively and actively cleared by the liver, an enormous advantage for cell therapies targeting the liver [23]. Therefore, performing the infusion of HA-coated hBTSCs in the portal vein—and even more so via the hepatic artery, if that proves feasible—could permit a greater number of cells to remain confined to the liver without increased extra-hepatic distribution. HA coating could play additional beneficial effects on cell therapy because HA can stimulate the expression of adhesion molecules, thus favoring the proliferation and engraftment of hBTSCs. Previous studies highlighted varied HA trophic effects in different cell systems [12, 13, 20, 21, 38, 42]. HA plays a key role in stabilizing and organizing the ECM by adjusting the adhesion and cell motility, mediating proliferation and differentiation as exemplified in the following examples. The authors demonstrated paracrine interactions between the implanted cells, the host ECM, and endothelial cells, and a proangiogenic effect of degradation of exogenous HA [12, 13, 20, 21, 38, 42].

A current limit of this study is the lack of a proof of performance of HA-coated hBTSCs in experimental models of liver diseases. However, the results obtained and previous observations by Carpino et al. [25] on the effects of freshly isolated hBTSCs infused in mice subjected to liver fibrosis/cirrhosis induced by carbon tetrachloride, demonstrating an improvement of serum liver biochemistry, guarantee further experiments where hBTSCs will be subjected to HA coating before cell transplantation.

Ectopic cell distribution has been shown to occur following transplants of stem cells into the liver, whether by vascular route or by direct injection, a finding of unknown importance clinically. Ectopic loci of transplanted uncoated cells occurred in all tissues in prior studies. Most studies assumed that the cells did not survive. However, more recent investigations have indicated that the ectopic sites are in most (if not all) tissues where the cells survived for months and could be identified using positron emission tomography [7]. In this study, we have evaluated both the lung and the kidney to test the occurrence of ectopic human cell emboli. Both the lungs and the kidneys appeared invariably normal with respect to histomorphological aspects. By IHC, ectopic distribution occurred only in the lungs and at levels comparable with that found for uncoated cells. This distribution did not occur in the kidneys, a second site known for HA clearance.

An especially advantageous aspect of using HAs to facilitate engraftment is that they are anti-inflammatory and entirely biocompatible. In prior studies with use of HAs for diverse forms of transplantations, they minimize fibrotic reactions and foster vascularization that facilitates engraftment [4]. Finally, no tumoral mass has been observed into the abdomen, liver, spleen, kidney, lungs, or derma at sacrifice, confirming results of prior reports in which the oncogenic potential of transplanted hBTSCs has been evaluated after months and has resulted in a complete absence of tumor formation [24, 25].

## Conclusion

We are proposing a simple, rapid, effective, and safe method to improve liver engraftment of transplanted cells for use in the cell therapy of liver diseases. HA-coated hBTSCs can be used immediately in clinical studies because HA is available in GMP conditions.

In summary, coating of hBTSCs with HAs resulted in a 4-fold increase (from ~3% to 11%) in engraftment even when delivered by the portal vein. It will be interesting to see whether HA coating increases the engraftment by an equivalent fold increase if injected into the hepatic artery, where prior findings of uncoated stem cells have indicated an engraftment of 20–25%. Clearly, this simple procedure of coating cells with HAs enables optimized engraftment of cells into the liver when delivered by a vascular route.

## Additional files

Additional file 1: Table S1. presenting end-point determination details.(DOC 30 kb)

Additional file 2: Table S2. presenting list of used antibodies and their application(s). (DOC 40 kb)

Additional file 3: Table S3. presenting positive and negative controls. (DOC 30 kb)

Additional file 4: Figure S1. showing human albumin gene expression in HA-coated hBTSCs (*dark gray columns*) (4.83 × 10^–7^ ± 3.95 × 10^–8^ vs 3.10 × 10^–6^ ± 3.02 × 10^–7^; *N* = 5; *p* < 0.05) and uncoated hBTSCs (*light gray columns*) (4.47 × 10^–7^ ± 7.22 × 10^–8^ vs 2.73 × 10^–6^ ± 3.48 × 10^–7^; *N* = 5; *p* < 0.05) had higher expression in differentiation conditions compared with self-renewal conditions. Data expressed as mean ± SD of *N* = 5 experiments. Human albumin gene expression in primary human hepatocytes, as a positive control, were markedly higher (3.64 × 10^0^ ± 2.02 × 10^–1^; *N* = 5; *p* < 0.01) than HA-coated hBTSCs and uncoated hBTSCs in differentiation and self-renewal conditions. (TIF 4903 kb)

Additional file 5: Word file presenting supplementary materials and methods. (DOC 30 kb)

## Abbreviations

BSA: Bovine Serum Albumin
CD: Cluster differentiation
CDH1: E-cadherin
cGMP: Current good manufacturing practice
DAPI: 4,6'-diamidino-2phenylidole
EpCAM: Epithelial cell adhesion molecule
ESC: Embryonic stem cell
FBS: Fetal Bovine Serum
HA: Hyaluronic acid
hBTSC: Human biliary tree stem cell
HDM: Hormonally defined medium
HepPar-1: Hepatocyte Paraffin 1
HpSC: Hepatic stem cell
IF: Immunofluorescence
iPS: Induced Pluripotent Stem
IHC: Immunohistochemistry
ITGβ: Integrin subunit beta
KM: Kubota’s Medium
PAS: Periodic Acid–Schiff
PD: Population Doubling
PDT: Population Doubling Time
PEG: Polyethylene glycol
RT-qPCR: Quantitative reverse-transcription polymerase chain reaction
SCID: Severely combined immunodeficient
