# Supplementary material for: Hyaluronan coating improves liver engraftment of transplanted human biliary tree stem/progenitor cells
Source: Stem Cell Res Ther. 2017 Mar 20;8:68. doi: 10.1186/s13287-017-0492-7 (PMC5360089; doi:10.1186/s13287-017-0492-7)
Supplement: Supplementary file 1 — presenting end-point determination details.(DOC 30 kb) [file 13287_2017_492_MOESM1_ESM.doc]

**Supplementary Table 1. End-point determination details**

The following genes of interest (GOI) were amplified using the primer pairs reported for each of them. The ratio of concentrations of GOI and the reference genes, namely, GAPDH for CDH1, CD44, ITGB1/4, and beta-actin for human and murine albumin, was assumed to be the GOI relative expression.

**Gene Id. Sequence Primers (5’ – 3’)**

CDH 1 E-Cadherin NM_004360.3 TCACAGTCACTGACACCAACGGA GGCACCTGACCCTTGTACGT

CD 44 Hyaluronan receptor NM_000610.3 TGCCGCTTTGCAGGTGTAT

GGCCTCCGTCCGAGAGA

ITGB 1 Integrin β 1 NM_002211.3 CAAAGGAACAGCAGAGAAGC ATTGAGTAAGACAGGTCCATAAGG

ITGB 4 Integrin β 4 NM_000213.3 CTGTGTTGCACGAGGGACATT

AAGGCTGACTCGGTGGAGAA

GAPDH NM_002046.3 AAGGTGAAGGTCGGAGTCAA

AATGAAGGGGTCATTGATGG

Human albumin NM_000477.5 AGAGGTCTCAAGAAACCTAGGAAA

GGTTCAGGACCACGGATAGA

Mus musculus albumin NM_009654.3 CGAGAAGCTTGGAGAATATGGA

CTTGGTGCCCACTCTTCCTA

Mus musculus beta-actin NM_007393.5 GGATGCAGAAGGAGATTACTGC

CCACCGATCCACACAGAGTA
