# Supplementary material for: Hyaluronan coating improves liver engraftment of transplanted human biliary tree stem/progenitor cells
Source: Stem Cell Res Ther. 2017 Mar 20;8:68. doi: 10.1186/s13287-017-0492-7 (PMC5360089; doi:10.1186/s13287-017-0492-7)
Supplement: Supplementary file 2 — presenting list of used antibodies and their application(s). (DOC 40 kb) [file 13287_2017_492_MOESM2_ESM.doc]

**Supplementary Table 2.** List of used antibodies and their application(s)

| **Name** | **Host / isotype** | **Source** | **Catalog#** | **Dilution** | **Application** |
| --- | --- | --- | --- | --- | --- |
| Hyaluronic acid | Rabbit IgG | Cloud-Clon Corp. | PAA182Ge01 | 1:100 | IF |
| Albumin | RabbitIgG | Abcam | ab108788 |  | ELISA |
| Anti-Human Mitochondria | Mouse IgG1 | Chemicon | MAB1273 | 1:200 | IHC |
| HepPar-1 | Mouse IgG1 | DAKO | M7158 | 1:50 | IHC |
| SOX17 | GoatIgG | R&D | AF1924 | 1:50 | IHC/IF |
| Mrp-2 | Mouse IgG2a | Abcam | ab3373 | 1:200 | IF |
| CD44 | Mouse IgG | Cell Signaling | 3570 | 1:50 | IHC/IF |
| αSMA | Mouse IgG2a | Dako | M8051 | 1:50 | FC |
| CD45 | Mouse IgG1 | BD | 561865 | 1:50 | FC |
| CD31 | Mouse IgG1 | DAKO | GA610 | 1:50 | FC |
| CD90 | RabbitIgG | Abcam | ab133350 | 1:50 | FC |
