# Supplementary material for: Hyaluronan coating improves liver engraftment of transplanted human biliary tree stem/progenitor cells
Source: Stem Cell Res Ther. 2017 Mar 20;8:68. doi: 10.1186/s13287-017-0492-7 (PMC5360089; doi:10.1186/s13287-017-0492-7)
Supplement: Supplementary file 3 — presenting positive and negative controls. (DOC 30 kb) [file 13287_2017_492_MOESM3_ESM.doc]

**Supplementary Table 3.** Positive and Negative Controls

| **ANTIGEN** | **METHODS** | **POSITIVE CONTROL** | **NEGATIVE CONTROL** |
| --- | --- | --- | --- |
| Albumin | Enzyme-linked immunosorbent assay (ELISA); RT-qPCR | Mature human hepatocytes | Mature cholangiocytes |
| human mithocondria | Immunohistochemistry /Immunefluorescence (IHC/IF) | Human liver | Mouse Liver |
| Hep-Par1 | IHC/IF | Human liver | Mouse Liver |
| human Albumin | IHC/IF | Human liver | Mouse Liver |
| Periodic Acid-Schiff (PAS) | IHC/IF | Liver | Hepatic stem/progenitor cells |
