# Supplementary figures and images for: Hyaluronan coating improves liver engraftment of transplanted human biliary tree stem/progenitor cells
Source: Stem Cell Res Ther. 2017 Mar 20;8:68. doi: 10.1186/s13287-017-0492-7 (PMC5360089; doi:10.1186/s13287-017-0492-7)

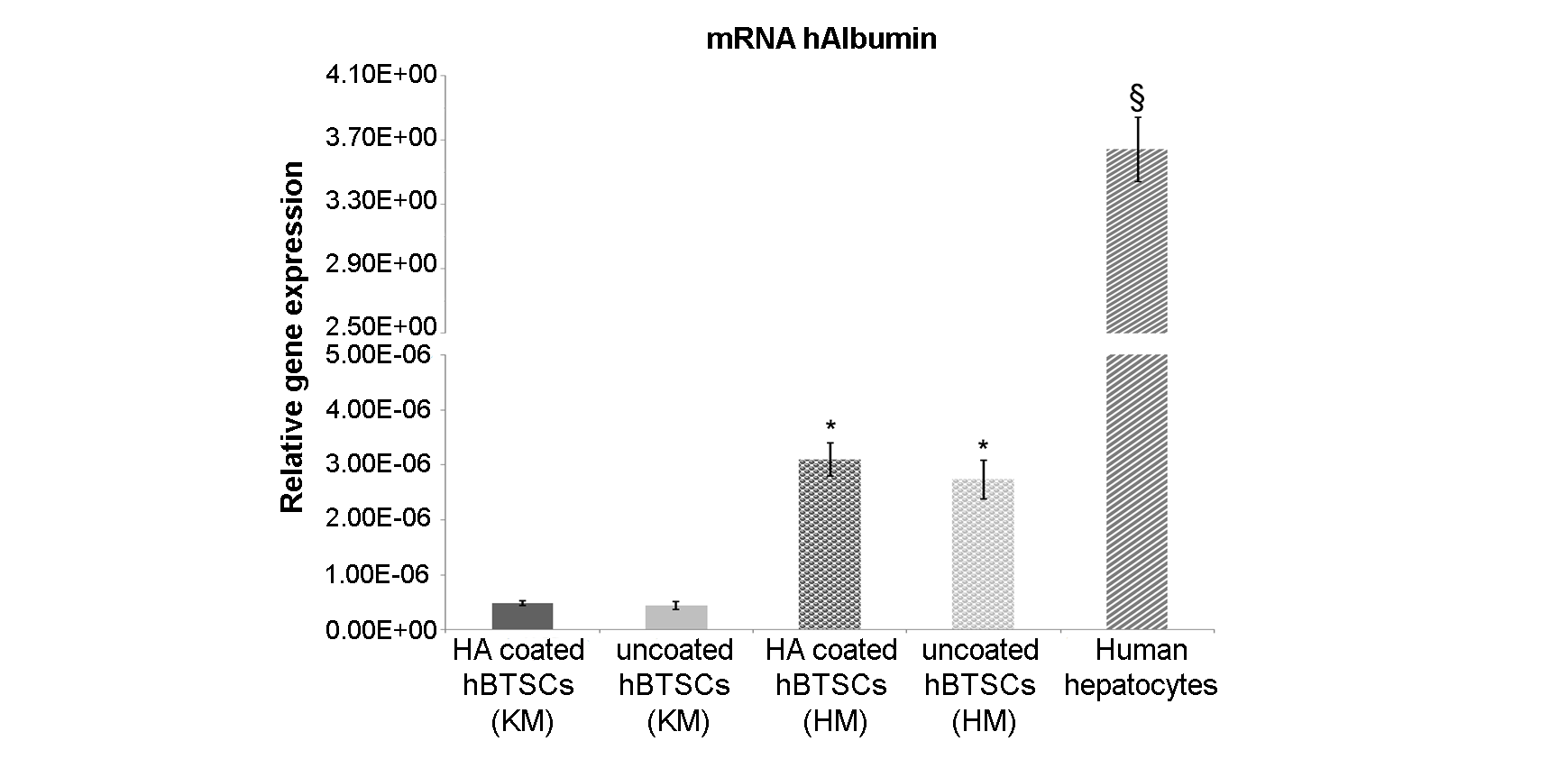

Supplement: Supplementary file 4 — showing human albumin gene expression in HA-coated hBTSCs (dark gray columns) (4.83 × 10^–7^ ± 3.95 × 10^–8^ vs 3.10 × 10^–6^ ± 3.02 × 10^–7^; N = 5; p < 0.05) and uncoated hBTSCs (light gray columns) (4.47 × 10^–7^ ± 7.22 × 10^–8^ vs 2.73 × 10^–6^ ± 3.48 × 10^–7^; N = 5; p < 0.05) had higher expression in differentiation conditions compared with self-renewal conditions. Data expressed as mean ± SD of N = 5 experiments. Human albumin gene expression in primary human hepatocytes, as a positive control, were markedly higher (3.64 × 100 ± 2.02 × 10^–1^; N = 5; p < 0.01) than HA-coated hBTSCs and uncoated hBTSCs in differentiation and self-renewal conditions. (TIF 4903 kb) [file 13287_2017_492_MOESM4_ESM.tif]
