# Supplementary material for: Hyaluronan coating improves liver engraftment of transplanted human biliary tree stem/progenitor cells
Source: Stem Cell Res Ther. 2017 Mar 20;8:68. doi: 10.1186/s13287-017-0492-7 (PMC5360089; doi:10.1186/s13287-017-0492-7)
Supplement: Supplementary file 5 — Word file presenting supplementary materials and methods. (DOC 30 kb) [file 13287_2017_492_MOESM5_ESM.doc]

**Supplementary data**

**Materials and Methods**

**SM.1. Differentiation Medium**

The serum-free Kubota’s Medium was supplemented with calcium (final concentration 0.6 mM), copper (10-12 M) and 20 ng/mLbFGF (Life Technologies # 13256029) and referred to as modified Kubota’s Medium (MKM). Hormonally defined media (HDM) for hepatocyte differentiation (HM) was prepared supplementing MKM with 7 µg/L glucagon, 2 g/L galactose, 1nM triiodothyronine 3 (T3, SIGMA T2877), 10 ng/mL Oncostatin M (OSM, SIGMA # o9635); 10 ng/mL epidermal growth factor (EGF, SIGMA # E9644), 20 ng/mL hepatocyte growth factor (HGF, SIGMA # H1404), and 1 µM dexamethasone.

**SM.2. Primary mature hepatocytes culture**

Primary mature hepatocytes were purchased from LONZA, Gampel, Valais (Switzerland) (LONZA #HUFS1M). Hepatocytes were cultured as indicated by LONZA using the indicate media: Hepatocyte Plating Medium (LONZA # MP250) and Hepatocyte Maintenance Medium (LONZA # MM250).

**Figure legend**

**Supplementary Figure 1**

Human albumin gene expression in HA-coated hBTSCs (dark grey columns)(4.83*10^-7^ ± 3.95*10^-8^ vs 3.10*10^-6^ ± 3.02*10^-7^; N=5; p<0.05)and uncoated hBTSCs (light grey columns)(4.47*10^-7^ ± 7.22*10^-8^ vs 2.73*10^-6^ ± 3.48*10^-7^; N=5; p<0.05)had higher expressionin differentiationconditions compared with self-renewalconditions. Data are expressed as the mean ± standard deviation (SD) of N=5 experiments. Human albumin gene expression in primary human hepatocytes, as a positive control, weremarkedly higher (3.64*10^0^ ± 2.02*10^-1^; N=5; p<0.01) thanHA-coated hBTSCs and uncoated hBTSCs in differentiation and self-renewal conditions.
